# Assessment of Job satisfaction of group of nurses in ava salamat entrepreneurs institute in Iran

**DOI:** 10.1016/j.dib.2018.04.043

**Published:** 2018-04-17

**Authors:** Mohammad Mirzabeigi, Ali Fardi, H. Raza Yousofi, Mahdi Norouzian, Mahdi Pariav, Saeid Lak, Majid Radfard, Abbas Ebadi

**Affiliations:** a In Entrepreneurship Management, Head of Entrepreneurship at the Ministry of Health and Medical Education, Tehran, Iran; b Ava Salamat Entrepreneurs Institute, Tehran, Iran; c Science and Research branch, Islamic Azad University Hamedan, Iran; d Department of Industrial Management, Central Branch, Islamic Azad University, Tehran, Iran; e Torbat Heydariyeh University of Medical Sciences, Torbat Heydariyeh, Iran; f Health Research Center, Baqiyatallah University of Medical Sciences, Tehran, Iran; g Abadan University of Medical Sciences, Abadan, Iran; h Behavioral Sciences Research Center, Faculty of Nursing, Baqiyatallah University of Medical Sciences, Tehran, Iran

**Keywords:** Job satisfaction, Ava salamat, Nurses

## Abstract

Global Health systems encounter increasing challenges, spread of health needs and economic constraints. Approximately, nurses are the major part of human resources working in health systems in all countries. Job dissatisfaction is one of the effective factors in nursing career exit. This study has been accomplished with purpose of determining nurses' job satisfaction in Ava Salamat Entrepreneurs Institute. This cross-sectional and descriptive research was performed in 2017. A random group of 533 nurses contributed in the study. A questionnaire was used for data collection, which included personal and career attributes, and level of job satisfaction as inputs. Data was collected over a period of three months. The Statistical Package for the Social Sciences (SPSS v22) software and descriptive statistical tests were utilized for the analysis. According to results, nurses job security was increased impressively, more than before they were employed in Ava Salamat Entrepreneurs Institute (about 62%), and they feel satisfied about their position more than before (77.1%) and have a desire to continue working for Ava Salamat Entrepreneurs Institute (75.4%). The results show that 62.9% of nurses were pleased for their prompt payment, and about 67% were dissatisfied with the proportion of their tasks and career hardship. Among those, 55.6% of nurses were satisfied by the professional support received from their managers and 51.4% of the nurses were satisfied with their image in the social profession.

**Specifications table**

**Table:** 

Subject area	Nursing and Health professions
More specific subject area	Nurses job satisfaction in Ava Salamat Entrepreneurs Institute
Type of data	Tables
How data was acquired	This research is cross-sectional and descriptive which has performed about nurses working throughout Iran hospitals in 2017 (15 universities of medical sciences in contract with Ava Salamat Entrepreneurs Institute)
Data format	Raw, Analyzed
Data source location	15 universities of medical sciences in contract with Ava salamat Entrepreneurs Institute (Ardebil, Alborz, Iran, Boushehr, Ahvaz, Hormozgan, Yazd, Zanjan, Save, Tabriz, Shoushtar, Ghazvin, Kordestan, Gilan, Maraghe)
Data accessibility	Data are included in this article

**Value of the data:**

• The job satisfaction difference between governmental and non-governmental nurses has been compared and analyzed for the first time. The comparison indexes include the behavioral disorders, paying salaries, benefits, welfare and educational facilities, for instance.
• By establishment of Ava Salamat Entrepreneurs Institute as non-governmental labor supplier, the job satisfaction has significantly been improved.
• Align with general policy of system in order to privatization and minifying the government.
• Increasing the interest in nursing majors will provide more job opportunities for nursing graduates.
• Impact of job satisfaction in growth of organization and staff productivity.

## 1 Data

**Fig. 1:**
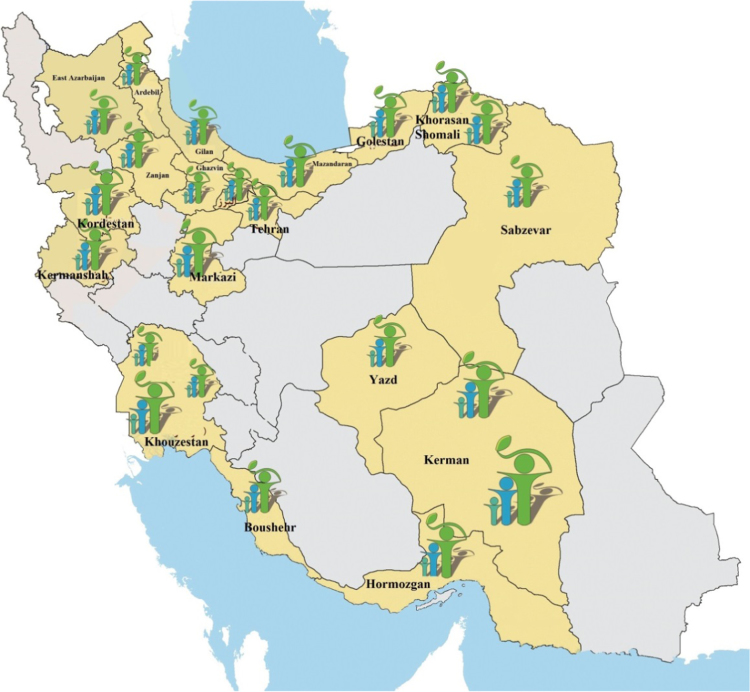
Location map of the study area.

The second section is job satisfaction measuring instrument prepared according to prior studies and remarks of nursing deputy executive managers. It has significant points in some questions. Those points are common concerns for nursing executive managers in Ministry of Health and Medical Education, guild organizations related to nursing association, and all nurses exclusive for a longtime (Prompt payment, career security, feeling the same positions with other governmental nurses, full premium payment of employer's share, appropriate work hours in comparison with other governmental nurses, etc.) Results of job satisfaction measurement are displayed in Table 3 [1], [2], [3], [4] (Fig. 1) (Table 1, Table 2)

**Table 1:** Personal attributes of 533 nurses working in 15 universities of medical sciences in contract with Ava salamat Entrepreneurs Institute.

Personal attributes		Number (%)
Age	25–30	309 (58%)
	30–39	202 (37.9%)
	40–50	22 (4.1%)
Gender	Woman	366 (68.7%)
	Man	167 (31.3%)
Marital status	Married	338 (63.4%)
	Single	195 (36.6%)

**Table 2:** Career attributes of nurses working in 15 universities of medical sciences in contract with Ava Salamat Entrepreneurs Institute.

Career attributes		Number (%)	Career attributes		Number (%)
Degree of education	Diploma	30 (5.6%)	Work experience	1–2	46 (8.6%)
	Associate	61 (11.4%)		3–5	291 (54.6%)
	Bachelor	418 (78.4%)		6–8	144 (27%)
	M.Sc. and higher	24 (4.5%)		9–11	32 (6%)
Workplace	General hospitals	190 (35.6%)		12–15	18 (3.4%)
	Teaching hospitals	331 (62.1%)		Higher 15	2 (0.4%)
	Etc.	12 (2.3%)	Department	pediatrics	41 (7.7%)
Career	Nurse	332 (62.3%)		women	111 (20.8%)
	Surgery room	60 (11.3%)		Surgery unit	76 (14.3%)
	Anesthetics	61 (11.4%)		Internal	65 (12.2%)
	Nurse assistant	76 (14.3%)		Emergency	65 (12.2%)
	Etc.	4 (0.8%)		Surgery room	133 (25%)
Professional position	Staff nurse	31 (5.8%)		Etc.	42 (7.9%)
	Nurse	388 (72.8%)	Shift work	Fixed morning	23 (4.3%)
	Nurse assistant	89 (16.7%)		Fixed afternoon	21 (3.9%)
	Etc.	25 (4.7%)		Fixed night	44 (8.3%)
				Rotational	445 (83.5%)

**Table 3:** Job satisfaction level of 533 nurses working in 15 universities of medical sciences in contract with Ava Salamat Entrepreneurs Institute. [11].

**Job satisfaction questions**	**Satisfied and highly satisfied number (%)**	**Dissatisfied and highly dissatisfied numbers (%)**
Do you satisfy with your career development opportunities?	310 (58.1%)	140 (26.3%)
Do you satisfy with your in-service training?	272 (51%)	167 (31.3%)
Do you satisfy with the proportion of your tasks and career hardship?	147 (27.5%)	341 (64%)
Do you satisfy with your prompt payment?	294 (62.3%)	198 (31.1%)
Do you satisfy with your revenue and benefits?	196 (36.8%)	292 (54.8%)
Do you satisfy with your job security in comparison with other staffs?	139 (26.1%)	302 (56.7%)
How do you evaluate your job security under contract with institute and before it?	330 (61.9%)	90 (16.9%)
Do you satisfy with the identical position between other governmental staffs and yours?	190 (35.7%)	289 (54.2%)
Do you satisfy with your workplace situation?	254 (47.7%)	179 (33.6%)
Do you satisfy with your insurance support?	389 (72.8%)	69 (12.9%)
Do you satisfy with given opportunities for using your skills?	361 (67.7%)	84 (15.7%)
Do you satisfy with your work hours?	253 (47.4%)	212 (39.7%)
Do you satisfy with your workload?	168 (31.5%)	273 (51.2%)
Do you satisfy with internal communication with your colleagues?	339 (63.6)	84 (15.8%)
Do you satisfy with rate of consideration for your ideas?	281 (52.7%)	119 (22.3%)
Do you satisfy with career support from your nursing managers?	296 (55.6%)	114 (21.4%)
How do you evaluate your image in the social profession?	274 (51.4%)	129 (24.2%)
Do you satisfy with the proportion of your duties and task types?	307 (57.6%)	109 (20.5%)
Do you satisfy with your position in the institute generally?	411 (77.1%)	53 (9.9%)
Do you satisfy with the continuity of cooperation with the institute under current situation?	402 (75.4%)	50 (9.4%)

## 2 Materials and methods

This research is cross-sectional and descriptive. It has performed about nurses working throughout Iran hospitals in 2017 (15 universities of medical sciences in contract with Ava salamat Entrepreneurs Institute). Research statistical population included nurses working in governmental and non-governmental hospitals all over Iran. Study entry requirements were having Iranian nationality and at least one year work experience.

According to prior studies sample size covered 10 percent of the population. This study was performed under the observance of ethical standards and license issued by ethics committee of Health Ministry nursing deputy. Study instrument was a questionnaire in two parts. First part was about nurses' personal and job attributes, such as age, gender, degree of education, marital status, workplace, professional position, and the second part was job satisfaction measuring instrument prepared by prior studies and remarks of nursing deputy executive managers [5], [6], [7], [8], [9], [10], [11], [12].

Entrepreneurs Institute. The authors express their appreciation to all representatives of the institute for distributing and collecting questionnaires and Managers of the Institute, Ali Mohammad Taghipour, Contract manager and Maryam Zahedi, Finance Director, Ali Dadgari (Nursing Consultant of Project), Esmaeel Izadi (Public Relations of Institute) and Mohammad Reza Gholamsim (Systems and methods expert of Institute), with the highest regards.
